# Starter culture growth dynamics and sensory properties of fermented oat drink

**DOI:** 10.1016/j.heliyon.2023.e15627

**Published:** 2023-04-25

**Authors:** Mary-Liis Kütt, Kaisa Orgusaar, Irina Stulova, Reimo Priidik, Dmitri Pismennõi, Helen Vaikma, Aili Kallastu, Aleksandra Zhogoleva, Indrek Morell, Tiina Kriščiunaite

**Affiliations:** a Center of Food and Fermentation Technologies, Mäealuse 2/4, Tallinn, 12618, Estonia; b Institute of Chemistry and Biotechnology, Tallinn University of Technology, Akadeemia tee 15, 12618 Tallinn, Estonia; c Department of Business Administration, School of Business and Governance, Tallinn University of Technology, Ehitajate tee 5, 12616 Tallinn, Estonia

**Keywords:** Vegan starter cultures, Dairy alternatives, Sensory profile, Starter consortium, Growth dynamics, Fermented oat drink

## Abstract

In the present study, an oat drink, a plant-based alternative to dairy products, was developed by fermenting the oat base with different vegan starter cultures. The desired pH below 4.2 was achieved in 12 h, regardless of starter culture used. Metagenomic sequencing revealed that *S. thermophilus* was the dominating species, ranging from 38% to 99% of the total microbial consortia*.* At lower pH values, population of *L. acidophilus*, *L. plantarum* and *L. paracasei* continued to increase in fermented oat drinks. Lactic acid was produced between 1.6 and 2.8 g/L. The sensory panel showed that all fermented oat drinks had a sour odor and taste. The volatile compounds identified belonged to the ketone, alcohol, aldehyde, acids, and furan classes. The concentration of the most preferred volatile components, such as diacetyl and acetoin, increased during fermentation. However, sensory evaluation showed that all samples were associated with cereals and not dairy in terms of taste and odor. Rheological analysis showed the formation of weak gel-like structures in fermented oat drinks. Overall, fermentation improved flavor and texture of the product. This study provides a broad overview of the oat drink fermentation process from the perspectives of starter culture growth, microbial consortium dynamics, lactic acid bacteria metabolism, and sensory profile formation.

## Highlights

• A 12 h fermentation with various vegan cultures lowered the pH below 4.2.
• The dominant species throughout the fermentation was *S. thermophilus.*
• The fermentation process was homofermentative and lactic acid was produced.
• Fermented samples were high in buttery and creamy volatiles.
• Fermentation improved and enriched the texture and flavor of the oat drink.

## Graphical abstract

**Figure:**
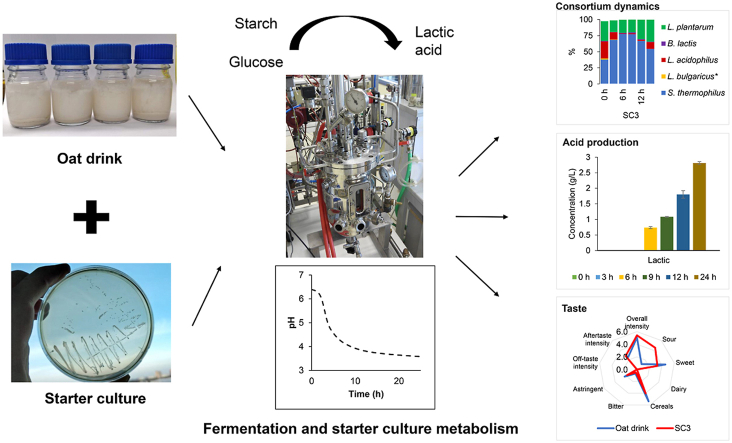

## 1 Introduction

Plant-based fermented drinks are the new reality towards a sustainable lifestyle. The world population will grow to 9.7 billion within five decades and there will not be enough animal-based food for all of us [1]. Plant-based foods also provide healthy and sustainable diet for mankind. To meet these needs, novel plant-based dairy alternatives that mimic animal-based foods must be developed.

Plant-based drinks, often called milk analogs, are gaining popularity every year. Cereals, legumes and nuts are the most preferred raw materials for making plant-based drinks [2,3]. Unfortunately, plant-based drinks often do not meet the requirements of traditional dairy products and require excessive fortification. Fermentation is a simple and natural way to improve the sensory, textural, and nutritional value of plant-based dairy alternatives. However, plant-based dairy alternatives are often not fermented, and both acid and live bacteria are added in the final stages of product preparation. Furthermore, if live cultures are used, very little is known about how traditional milk-derived bacteria behave and adapt to plant materials. Currently, we still lack information on culture growth kinetics, consortium composition, and flavor and aroma formation of plant-based fermented drinks. In order for bacteria to succeed, we need to understand how different starter cultures affect plant-based fermented drinks.

In our article, we decided to focus on oat drink because the consumption of oat drinks is a growing trend. Oats are healthy and less allergenic compared to soy or nut-based drinks. Oats grow in cool, moist conditions and in sandy loam to heavy clay soils with good drainage, which helps to cultivate oats in regions (e.g. Eastern Europe, Scandinavia, North America) where soy or nut cultivation could not be applicable due to weather and agricultural conditions [4].

Oats are rich in starch, protein, fiber (beta-glucans), antioxidants, vitamins, and healthy fats, with the higher protein and lipid content than other cereals [3,5]. The presence of starch turns the oat-based matrix into a gel. Enzymatic hydrolysis with α-amylase or sequential hydrolysis with α- and β-amylase to hydrolyze starch to glucose and shorter chain polysaccharides must be performed to maintain the fluidity of the beverage [3]. 80% of oat protein is globulins with an isoelectric point between 4 and 5. Fermented oat beverage often reaches this low pH concentrations, which causes protein aggregation and reduces the acceptance of the fermented product due to chalky and sandy mouthfeel [6]. However, nutritionally, oat proteins have a higher lysine content than wheat, which is the main limiting amino acid in cereals [7]. Oats also contain high amounts of unsaturated essential fatty acids, such as oleic acid (18:1) and linoleic acid (18:2), which have a significant impact on nutritional quality. Unfortunately, oats also contain a considerable amount of lipases, which can cause rancidity during oat processing [8].

Fermentation is one way to reduce the shortages of raw materials and improve the shelf-life, nutrition, and flavor of the product. Microorganisms involved in fermentation produce enzymes, vitamins such as folates, short chain fatty acids, amino acids, bacteriocins and exopolysaccharides, thereby improving the nutritional value, sensory properties, and shelf-life of the product. For example, riboflavin and folate are found in higher concentrations in fermented oat drink than in unfermented oat drink [9]. Short chain fatty acids produced by LAB increase the solubility of bioavailable calcium and enhance the synthesis of vitamins and bioactive peptides [10]. Fermentation also improves the appearance of plant-based dairy alternatives. Due to the production of organic acid and a decrease in pH, the effect on phenols has been shown to lighten the colour of the fermented product compared to unfermented oat beverage [11].

Lactic acid bacteria (LAB) are often used to positively manipulate plant-based dairy alternatives. To improve the aroma profile, *L. plantarum* NCIMB 8826 has been used in oat, wheat, barley, and malt matrices. Different volatile compounds such as esters, alcohols and aldehydes were produced during the growth, with each cereal broth having a unique volatile compound profile [12]. When traditional LAB strains were combined with *L. rhamnosus* LGG^R^ to ferment soy, oat, and coconut substrates, acetoin levels increased, and acetaldehyde decreased in the presence of LGG^R^ in all three bases. Oat samples additionally fermented with LGG^R^ demonstrated preferred notes, such as sourness, lemon, and fruity flavors. Gel firmness in coconut samples was improved in the presence of LGG^R^, indicating that the addition of *L. rhamnosus* improves the textural and sensory perception of fermented plant-based dairy alternatives [13]. *L. plantarum* LP09 strain was used to ferment the oat flake beverage. Oat base fermentation increased polyphenols availability and antioxidant activity (25% and 70% higher, respectively). Additionally, sensory evaluation showed that the fermented oat flake drink has characteristics of a yogurt-like beverage, enhancing the overall intensity of odor and flavor compared to the unfermented control [11]. To conclude, plant-based beverages will play a major role in future diets, and the fermentation of these matrices by LAB needs to be thoroughly investigated.

In this study, we combined various analysis and analytical tools to understand how starter cultures affect the oat drink during fermentation. The novelty of the work lies on the complex overview, where the growth dynamics of the starter culture are monitored from chemical-, physical-, metabolic- and sensory aspects. For the first time, isothermal microcalorimetry and 16 S metagenomic analysis are combined to evaluate the starter culture properties and composition in a fermented oat drink. This knowledge provides a detailed overview of what novel food manufactures need to consider when formulating and developing plant-based dairy alternatives.

## 2 Materials and methods

### 2.1 Materials

#### 2.1.1 Raw materials and chemicals

Veski Mati whole grain oat flakes were purchased from a local retailer. α-Amylase Fungamyl® 800 L (Fungamyl), α-amylase BAN® 480 L (BAN), and glucoamylase AMG® 300 L (AMG) were provided by Novozymes (Bagsvaerd, Denmark). For sugar analysis, D-(+)-Raffinose pentahydrate (Sigma-Aldrich, P/NR0250-25G), D-(+)-Maltose monohydrate (Sigma-Aldrich, P/N M2250-1 KG), D-(+)-Glucose (Sigma-Aldrich, P/N G7528-1 KG), were represented as marker compounds. For organic acid analysis, acetic acid (Honeywell, Fluka P/N 965,092), butyric acid (Sigma-Aldrich, P/N B1030500-500 mL), citric acid (Sigma-Aldrich, P/N 251,275-100G), formic acid (Supelco, P/N 5,330,020,050), isobutyric acid (Sigma-Aldrich, P/N l1754-500mL), isovaleric acid (Acros Organics, P/N AC156690100), lactic acid (Sigma-Aldrich, P/N L7022-10G), malic acid (Sigma-Aldrich,P/N M6413-25G), propionic acid (Sigma-Aldrich, P/N P1880-100G), succinic acid (Sigma-Aldrich, P/N 14,079-250G), valeric acid (Alfa-Aesar, P/N A16238. AP) were used as standards. For free amino acid analysis amino acid standard (Waters Corporation, WAT088122) + 3 additonal amino acids: l-asparagine (Serva, P/N 14,110), l-tryptophan (Serva, P/N 37,422) and l-glutamine (Sigma-Aldrich, G-3126) were used. For volatile compounds analysis in GC-MS, 4-methyl-2-pentanol (Sigma-Aldrich 109,916-25 mL) was used as internal standard.

#### 2.1.2 Starter cultures

Commercial vegan starter cultures were used to ferment the oat drink. Vegan starter cultures states that the lactic acid bacteria in the culture mix are produced dairy free, on plant-based medium and are suitable for plant and/or vegetable-based products. The products fermented with vegan starter cultures are suitable for vegans. Starter cultures are abbreviated SC1 to SC4 throughout the article. SC1 contains *Streptococcus thermophilus* (*S. thermophilus*) and *Lactobacillus delbrueckii* spp. *Bulgaricus* (*L. bulgaricus*). SC2 contains *S. thermophilus*, *L. bulgaricus*, *Lactobacillus delbrueckii* spp. *Lactis* (*L. lactis*), *Bifidobacterium lactis* (*B. lactis*) and *Lactobacillus acidophilus* (*L. acidophilus*). SC3 contains *S. thermophilus*, *L. bulgaricus*, *B. lactis*, *L. acidophilus* and *Lactobacillus plantarum* (*L. plantarum*). SC4 contains *S. thermophilus*, *L. bulgaricus*, *B. lactis*, *L. acidophilus* and *Lactobacillus paracasei* (*L. paracasei*). The manufacturer did not provide information on the proportion of individual bacterial species in the starter cultures.

### 2.2 Fermented oat drink preparation

To prepare 1 L of oat drink, 160 g of oat flakes were weighed and washed for 10 s under cold running water. 840 mL of water was added to the flakes and homogenized (Polytron PT MR 2100, Kinematica, Switzerland) for 30 s. To increase the enzyme activity, CaCl_2_ was added at a concentration of 0.01% (w/v oat base). Hydrolysis was performed with α-amylase (Fungamyl® 800 L or BAN 480 L) at a concentration of 0.88% (w/w oat flakes) and glucoamylase (AMG® 300 L) at a concentration of 0.4% (w/w oat flakes). The drink was hydrolyzed in a water bath (Julabo TW8, Julabo Labortechnik GmbH, Germany) for 60 min, ensuring +55 °C inside the liquid. After hydrolysis, the drink was strained through a muslin cloth and the solids were separated from liquid. The filtered drink was reheated to deactivate the enzymes in a water bath, where the internal temperature was maintained +85 °C for 15 min. The oat drink (enzymatically pretreated with Fungamyl) was cooled to +40 °C and divided into four aliquots. Each aliquot was inoculated with a commercial starter (SC1, SC2, SC3 and SC4). The freeze-dried culture was resuspended in sterile 0.85% saline and the inoculum concentration were 10 DCU. Fermentation was carried out for 24 h at +40 °C. For the various analysis, samples were collected and stored either at +4 °C (for sensory evaluation, NGS, rheological and titratable acid analysis) or −20 °C (for sugar, organic acid, free amino acid, and GC-MS analyses).

### 2.3 Isothermal microcalorimetry

Isothermal microcalorimetry can be used to monitor the heat flow produced by the microorganisms in real-time. This provides a continuous real-time signal proportional to the heat generated during bacterial growth and metabolism [14]. A TAM III 24-channel isothermal microcalorimeter (TA Instruments, New Castle, DE, USA) was used. Each 3 mL ampoule was filled with 2 mL of inoculated oat drink. The heat flow was monitored for 24 h at +40 °C. Data analysis was performed in Microsoft® Excel® (Version 2204 Build 16.0.15128.20240) by plotting power-time curves describing the heat release during the investigated process. The maximal specific growth rate μ_max_ (h^−1^) was determined as the slope of heat production (Q) of the exponential growth phase over time (t). The maximum heat flow produced P_max_ (μW/mL), and the time maximal heat flow obtained t_Pmax_ (h) were obtained from the power-time curves. The value P_max_ characterizes the maximal heat rate production and reaching P_max_ can be considered as the end of the exponential growth phase. The heat release during the exponential growth phase Q_exp_ (J/mL) and Q_tot_ (J/mL) during the entire growth were determined from the area under the power-time curves.

### 2.4 pH measurement

iCinac (AMS Alliance, Rome, Italy) is a multi-channel system for real-time monitoring of pH, temperature, and redox potential. For analysis, 104 g of the sample was placed in an autoclaved 100 mL bottle with a hole in the cap for the sensor, placed in a +40 °C water bath (Julabo EH, Julabo Labortechnik GmbH, Germany) and connected to an electrode. Changes in pH were monitored for 24 h. Data analysis was performed in *Microsoft Excel* by plotting pH curves.

### 2.5 Starter culture consortium analysis

#### 2.5.1 Microbial cell separation and genomic DNA extraction

To isolate bacterial cells from fermented plant residues, pellet from approximately 14 mL of fermented oat drink was resuspended in 10 mL of sterile 0.85% NaCl solution and centrifuged at 750×*g* (Hettich ROTANTA 460 R, fixed angle rotator) for 5 min at +6 °C. To pellet the microbial cells, the supernatant was transferred to a new 50 mL tube and centrifuged at 10,000×*g* for 15 min at +6 °C (Hettich ROTANTA 460 R, fixed angle rotator). The pellet was washed in 1 mL sterile 0.85% NaCl solution, divided into three aliquots (approximately 400 μL each) and centrifuged at 10,000×*g* for 10 min at +6 °C (Thermo scientific MicroCL 21 R). The supernatant was aspirated, and the pellet containing the microbial cells were stored at −20 °C until gDNA extraction.

Microbial cells precipitate from 400 μL aliquots (1/3 from all separated cells, approximately 100 mg of cells) were subjected to gDNA extraction according to the Quick-DNA™ Fungal/Bacterial Miniprep Kit protocol (ZR, Zymo Research, Irvine, CA, USA). The samples were thawed at room temperature for 10 min before the initiation of gDNA extraction. The quantity of the extracted gDNA was measured with a Qubit™ 4 Fluorometer (Thermo Fisher Scientific, Waltham, MA, USA) using the dsDNA BR Assay Kit (Thermo Fisher Scientific).

#### 2.5.2 16 S library preparation, next generation sequencing and data processing

Amplicon libraries targeting the 16 S rRNA gene V4 hypervariable region by primer pair 515 F/806 R were prepared according to Illumina's dual indexing system. Multiplexed and normalized libraries were sequenced with iSeq100 Sequencing System (Illumina, San Diego, CA, USA) using iSeq 100 i1 Reagent and 2 × 150 cycles paired-end sequencing protocol. Previous activities were performed as published before by Kazantseva et al., [15]. The sequencing data was analyzed by an open-source BION-meta package (https://github.com/nielsl/mcdonald-et-al) according to the author's instructions [16,17].

### 2.6 Chemical, textural, and sensory analysis

#### 2.6.1 Sugars and organic acid analysis

Enzyme-treated and fermented oat drink samples were centrifuged at 14,000×*g* for 20 min at room temperature (Hettich ROTANTA 460 R, fixed angle rotator). The supernatant was filtered through a 3 kDa molecular weight cut-off filter (Amicon® Ultra-0.5, Merck KGaA, Germany) and diluted with 2 parts of ultrapure water before analysis. Concentrations of sugars (D-(+)-Raffinose pentahydrate and D-(+)-Maltose monohydrate, represented as a marker compound), organic acids and D-(+)-Glucose were measured with a high-performance liquid chromatography (HPLC) system (Alliance 2695 system, Waters Corp., Milford, MA, USA), using a BioRad Aminex HPX-87C (for sugars) or BioRad Aminex HPX-87H (for organic acids) columns (7.8 × 300 mm, 9 μm particle size) (Bio-Rad Laboratories, Inc., CA, USA). A BioRad Micro-Guard Cation C guard column (4.6 × 30 mm, 9 μm particle size) with isocratic elution of ultrapure water at a flow rate of 0.6 mL/min at +85 °C was used for sugar analysis. H guard column (4.6 × 30 mm, 9 μm particle size) with isocratic elution of 5 mM H_2_SO_4_ at a flow rate of 0.6 mL/min at +35 °C was used for organic acid analysis. Waters 2414 refractive index detector was used for the detection and quantification of substances, which was paired with a Waters 2487 Dual Absorbance Detector for organic acid analysis.

##### 2.6.1.1 Titratable acidity

Titratable acidity, reported as lactic acid, was measured with a DL22 Food and Beverage Analyzer (Mettler Toledo, Switzerland). 5 g of sample was mixed with 45 g of distilled water, mixed until homogeneous and titrated with 0.1 N NaOH. The results were calculated as % of lactic acid using Eq. (1).

(1) $$\%\;lactic\;acid=\frac{mL∙N∙90∙100}{V∙1000}$$

Where: mL – NaOH usage for a sample in milliliters.

N – the normality of NaOH (0.1)
V – sample volume (5 mL)

#### 2.6.2 Free amino acid analysis

Fermented oat drink samples were centrifuged at 14,000×*g* for 20 min at room temperature (Hettich ROTANTA 460 R, fixed angle rotator). The supernatant was filtered through a 3 kDa molecular weight cut-off filter (Amicon® Ultra-0.5, Merck KGaA, Germany) and diluted with 2 parts of ultrapure water before analysis. Prior to injection, free amino acids were derivatized with AccQ•Fluor Reagent (Waters Corp., MA, USA) according to the manufacturer's procedure. Analysis of free amino acids was performed on an ultra-performance liquid chromatography (UPLC) system (Acquity UPLC; Waters Corp., MA, USA), including a binary solvent manager, a sample manager, and a photodiode array detector (PDA), controlled by Waters Empower™ 3.0 software (Build 3471, Waters Corp., MA, USA). Separations were performed on Waters Acquity UPLC AccQ•Tag Ultra Column (2.1 × 100 mm, 1.7 μm particle size) operated at +55 °C. The injection volume was 1.5 μL, the amino acids were eluted at a flow rate of 0.3 mL/min, and absorbance was recorded at 260 nm. The running time was 25 min. Empower software (Waters Corp., MA, USA) was used for data processing.

#### 2.6.3 Rheological analysis

Dynamic oscillatory measurements were carried out at +22 °C using a Physica Modular Compact Rheometer MCR 301 (Anton Paar GmbH, Graz, Austria) equipped with a Peltier temperature control unit *C*-PTD200 and a coaxial cylinder measuring system CC27 (outer and inner diameters 28.92 and 26.66 mm, respectively). Amplitude sweep was performed varying the strain from 0.01 to 100% at a constant frequency of 1 Hz. Frequency sweep was performed from 0.01 to 10 Hz at a strain value of 0.1%, staying within the linear viscoelastic (LVE) range. The storage (G′) and loss (G″) module were measured and plotted in double logarithmic scale against frequency and amplitude, respectively. The limit of the LVE range (γ_L_), the yield stress (τ_y_), and the storage modulus within the LVE range (G’_LVE_) were determined from the amplitude sweep. The slope of G′ vs frequency (Δ log G’/Δ log f) was calculated from the double logarithmic plot of frequency sweep using Rheoplus/32 V^2^.66 software (Anton Paar GmbH).

#### 2.6.4 Volatile compound analysis

Identification and quantification of volatile compounds was performed using gas chromatograph system (2030; Shimadzu, Kyoto, Japan) equipped with mass spectrometer (8050NX Triple Quadrupole; Shimadzu, Kyoto, Japan). A ZB5-MS column (30 m length × 0.25 mm i. d. × 1.0 μm film thickness; J&W Scientific, Folsom, CA, USA) was used with helium as a carrier gas at linear velocity of 35 cm s^−1^. The oven was programmed to ramp up from +40 °C at a rate of 7.5 °C/min to a final temperature of +280 °C with an additional holding time of 4 min (total run time 36 min). Mass spectra were obtained at an ionization energy of 70eV, detector voltage 1 kV and with Q3 scan range of *m*/*z* 35–250. Non-targeted identification of volatile compounds was carried out using GCMS solution 4.52 software (Shimadzu, Japan) and retention indices (RI) calculated with n-alkanes. The identification of the compounds was verified by comparing experimental retention indices to NIST17 and FFNSC4 spectral libraries. Semi-quantitative evaluation using the internal standard (4-methyl-2-pentanol; 20 ppb) was performed to semi-quantify identified volatile compounds (in ISTD ppb-equivalents).

#### 2.6.5 Sensory analysis

The sensory analysis was carried out by the sensory panel of Center of Food and Fermentation Technologies in a quiet room in accordance with ISO standard 8589:2007. The analysis was conducted by nine trained assessors (average age 32.8 ± 7.5) with previous experience in evaluating fermented plant-based dairy alternatives and fermented dairy products. All participants from a pool of highly trained evaluators in the sensory panel gave written consent to take part in the experiment. Participants were informed in advance of the purpose and the procedures of the study. Participants were assured of the confidentiality of their data. Taking part in the given study was voluntary and one could withdraw from the test at any time. Participants were in good health and had no known allergy to the components. Institutional approval for the research is not available due to Estonian requirements for human research.

A separate training session was carried out with selected samples prior the analysis, which familiarized assessors with the products and attributes for the assessment. The samples were kept refrigerated (+4 °C) until serving in 40 mL plastic cups coded with random three-digit numbers. Order of fermented drinks was followed by Williams Latin Square design. The appearance, odor, taste, and texture of samples were evaluated at 10-point scale ranging from 0 to 9 with anchor points (i.e., “0" - none; “1" - very weak; “5″ moderate; “9" - very strong). Panel members were encouraged to use water and crackers for palette cleansing. Samples were evaluated in three parallels, in total of two separate sessions. Unfermented oat drink was used as reference in both sessions. Assessors also had at least 2-h break between sessions to reduce sensory fatigue.

### 2.7 Statistics

Statistical analysis was performed in R version 4.0.2 (R Foundation for Statistical Computing, Vienna, Austria). Package ‘stats’ 4.0.2 was used for calculating principal components, performing ANOVA and Tukey Honest Significant Differences test at 95% family-wise confidence level.

Principal component analysis (PCA) for volatile compounds was performed using “prcomp” R function and visualized with R package “ggplot2” version 3.3.0. Sample clusters on PCA biplot were shown by 95% confidence ellipses. Variables were centered and scaled. Data represents each sample in the form of mean of biological replicates (n = 3).

## 3 Results and discussion

### 3.1 Oat drink preparation

#### 3.1.1 Enzymatic treatment

**Fig. 1:**
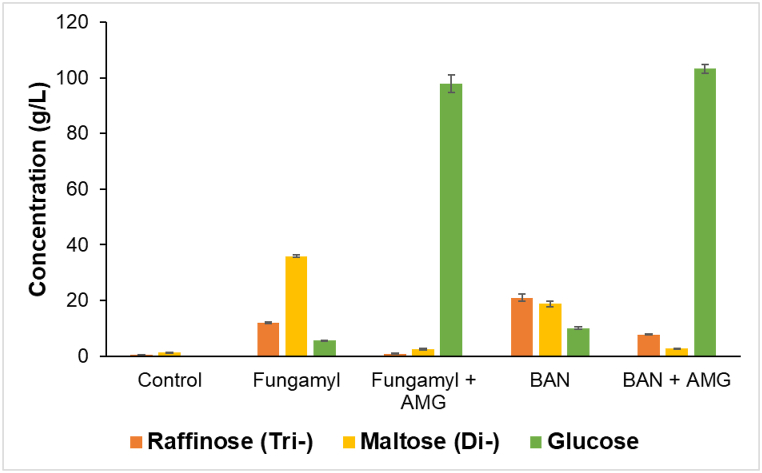
Sugar concentration (g/L) after enzymatic treatment of oat drink. Tri- and disaccharides are indicated as sum, where either raffinose or maltose was used as markers, respectively. AMG, BAN and Fungamyl are abbreviations for corresponding enzymes (see Materials and methods). Data represents each sample in the form of mean of biological replicates ± SD (n = 3).

The pretreatment of oat drink with different amylases to hydrolyze starch and obtain sugars for both, the fermentation process and the overall flavor profile of the final product are shown in Fig. 1. The addition of AMG increased the glucose concentration almost 10-times compared to the treatments without AMG. In the BAN treatment, the concentration of trisaccharides (20 g/L) was the highest following disaccharides and glucose. Fungamyl alone increased the disaccharide concentration to 35.86 g/L after 1 h of incubation. Trisaccharides and glucose were in similar concentration. Fungamyl was chosen for the final production of the oat drink as it resulted in a suspension with a nice creamy texture (compared to other enzymes that resulted in an unstable texture), and a slightly sweet and not too sugary taste (data not shown). High glucose concentration was also not preferred due to osmotic stress, which can inhibit lactic acid bacteria fermentation [18].

#### 3.1.2 Duration of hydrolysis

**Fig. 2:**
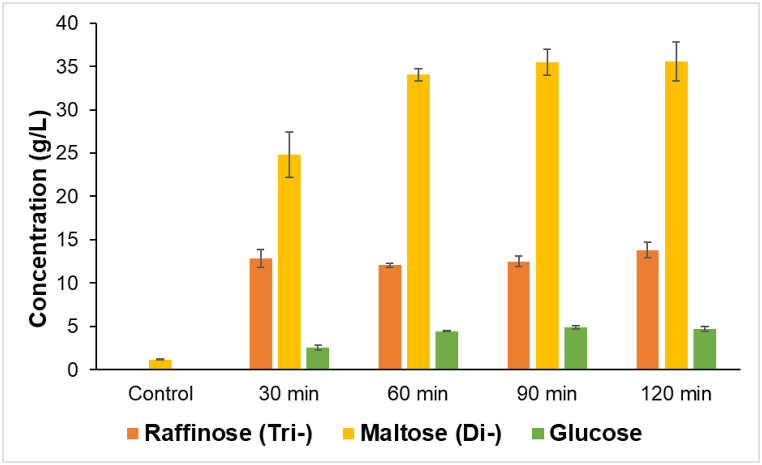
Sugar concentration (g/L) by different duration of Fungamyl enzyme treatment. Tri- and disaccharides are indicated as sum, where either raffinose or maltose was used as marker, respectively. Data represents each sample in the form of mean of biological replicates ± SD (n = 3).

The optimal time of enzymatic hydrolysis was determined at four timepoints and the results are shown in Fig. 2. The concentrations of trisaccharides, disaccharides and glucose were measured after 30, 60, 90 and 120 min of treatment with Fungamyl. Hexoses and disaccharides are the most favorable carbon source for lactic acid bacteria [19]. Since no significant increase in the concentration of tri- and disaccharides or glucose was observed after 60 min of hydrolysis, then 1 h enzyme treatment was considered sufficient for further experiments.

### 3.2 Fermentation of oat drink

#### 3.2.1 Dynamics of fermentation process

**Fig. 3:**
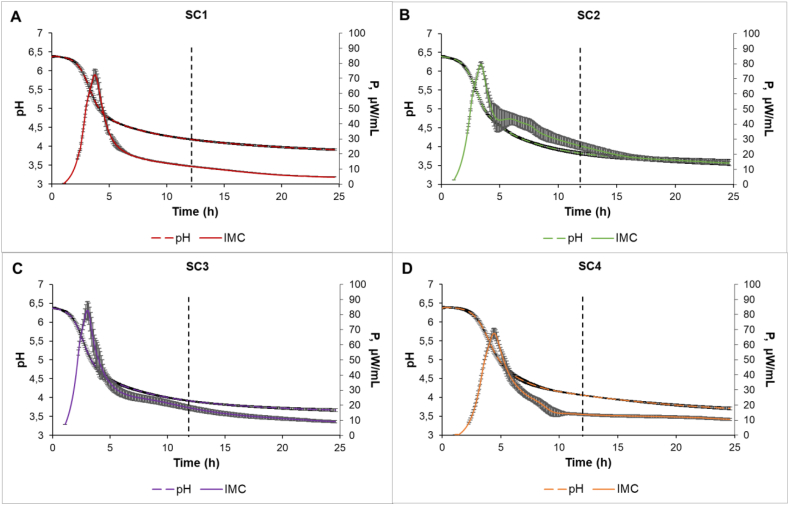
Heat flow curves (marked as IMC) and pH curves (marked as pH) of each starter culture for 24 h. Heat flow and pH curve for SC1 is shown on graph A, for SC2 on graph B, for SC3 on graph C and for SC4 on graph D. Curves from IMC are power time curves (P) with μW/mL values. The vertical dotted black line indicates hour 12, where extra samples for sensory evaluation, rheology and GC-MS analysis were collected. Data represents each sample in the form of mean of biological replicates ± SD (n = 3).

Based on specification sheets for commercial starter cultures, plant-based dairy alternatives should be fermented for 8.5–10 h to reach a pH of 4.6. However, for this experiment, the process was followed for 24 h to observe the dynamics of a longer fermentation (Fig. 3). In 24 h, the most acidified oat drink with a pH of 3.85 was obtained in the SC2 starter culture. In this experiment, the optimal fermentation time was 12 h, as a pH < 4.6 would result in a more acidic taste pallet in the final product, comparable to kefir with a pH of 4.2. At this time point, the SC1 culture had the mildest acidity with a pH of 4.19, followed by the more acidic SC4 with a pH of 4.06, then SC3 with a pH of 3.91 and SC2 with a pH of 3.82. Luana et al. [11], showed that fermentation of oat beverage with *L. plantarum* LP09 achieved a pH of 4.2 in 8 h. The observation was in correlation with a current study where a pH of 4.2 was reached already in 7.3 h with SC3, which also contains *L. plantarum*. If the desired optimal pH were 4.6, which is more comparable with yoghurt, then SC3 and SC2 reached pH 4.6 in ∼4.5 h, while SC1 and SC4 reached in ∼6 h. The degree of acidification of the oat drink appeared faster than the specification sheets of the starter culture stated. However, products such as yoghurt have a shorter shelf-life at pH 4.6 than at pH 4.2 or lower. Karagül-Yüceer et al. [20], showed that pH affected the viability of contaminating bacteria. *E. coli* survival at pH 5 was 21 days, while at low pH (4.2) there was no survival at day 21. After 12 h of fermentation (samples collected to sensory evaluation, rheology, and GC-MS study) drinks with SC3 and SC2 were only 0.1 pH points apart, showing that these two cultures acidify quite similarly.

The lag-phase before fermentation varied from 1 to 1.5 h depending on the starter culture. A similar lag-phase was observed for heat power time curves. While SC3 and SC2 showed the fastest acidification, the maximal specific growth rate (μ_max_) based on biomass heat production was highest for SC1 (Supplementary materials - Table S1) with 1.66 h^−1^. The maximal specific growth rates of SC2 and SC3 were 1.55 h^−1^ and 1.53 h^−1^, respectively. The lowest μ_max_ of 1.40 h^−1^ was observed for SC4. However, the time where exponential growth ended (P_max_) was fastest with SC3 at 3.03 h (t_Pmax_). The slowest grower, as already indicated by the maximal specific growth rate, was SC4, achieving the majority of the biomass in 4.4 h. Still, the maximal biomass (Q_tot_) was produced by SC2 reaching the total heat production of 2.19 J/mL. SC2 also had the lowest pH, suggesting greater acid tolerance while maintaining metabolic activity at low pH. The next highest total heat production was obtained with SC3, showing 1.94 J/mL. Interestingly, the slowest grower, SC4, produced more biomass and remained metabolically active longer than SC1, which initially had the highest maximal specific growth rate but then rapidly slowed down. The maximal total heat production for SC4 and SC1 was 1.63 J/mL and 1.29 J/mL, respectively.

The heat curves show (Fig. 3) that all three starter cultures except SC1 had two growth phases. It may be that SC1 has only two species in the consortium, while the other starter cultures have five species in the consortium. The second slower growth phase may indicate several environmental changes. First, essential substrates were depleted, and the starter culture had to switch on another source of nutrients. However, this scenario is the least likely, while the carbon source was unlimited (Supplementary materials – Fig. S.1). A second and more probable scenario was that whatever limiting property appeared in the medium, then either the dominating culture had to switch metabolism or slowly growing species started to take the culture over. The most probable cause was a drop in pH between 5 and 10 h. The limiting effect of pH caused the termination of the exponential growth phase. The pH curves show an exponential acidification phase within two to 5 h after the pH reaches the inhibitory range and the dominating bacteria or the whole consortium needs to change their metabolism to adapt to the new conditions.

#### 3.2.2 Starter culture consortium composition

**Fig. 4:**
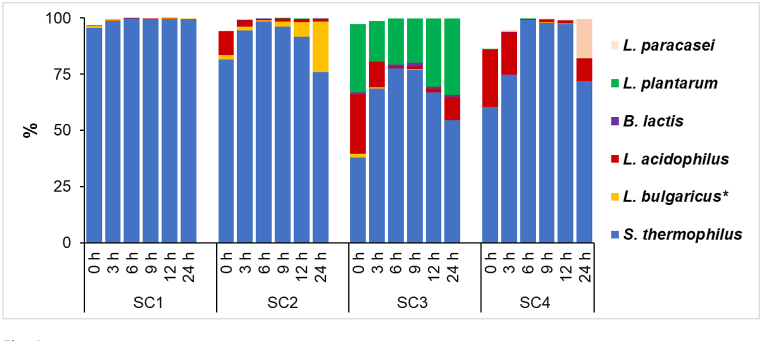
Dynamics of starter culture consortium during fermentation process. Data is represented as bacterial proportion in percentages using 16 S rRNA gene next generation sequencing method. In SC2, *Lactobacillus delbrueckii* ssp. *Bulgaricus* (*L. bulgaricus*) and *Lactobacillus delbrueckii* ssp. *Lactis* (*L. lactis*), are marked as L. bulgaricus*. Data represents each sample in the form of pooled biological replicates (n = 3).

The acidification curves as well as the heat power curves showed the shift and changes in the metabolism of the starter cultures during fermentation. To further investigate dynamic shifts between starter cultures, 16 S rRNA sequencing was carried out every 3 h for up to 12 h, with the next and final sampling point after 24 h of fermentation. Fig. 4 shows the relative abundance of starter species from whole consortia. Before the oat drink was fermented, the native bacteria formed 2.6–13.9% of the total population. The native microbiota was gradually outcompeted by starter cultures and had decreased to 0.4% after 6-h of fermentation. The native microflora was dominated by *Bifidobacterium animalis*, *Lactobacillus gallinarum*, *Streptococcus salivarus*, *Streptococcus suis,* and *Pseudomonas azotoformans*, which can be found on the surface of cereals or in milk [[21], [22], [23]].

All four starter cultures show that *S. thermophilus* was the dominating species throughout the fermentation. The main reason for the dominance of *S. thermophilus* is that this species prevailed in starter culture right from the beginning – in SC1: 95.7%, SC2: 81.6%, SC3: 38% and in SC4: 60.3% of the total consortium.

The SC1 specification states the presence of two species: *S. thermophilus* and *L. bulgaricus*. The 0-h sampling point shows that *S. thermophilus* is the dominating species, while *L. bulgaricus* was less than 1%. *S. thermophilus* dominated throughout the fermentation, showing almost monocultural fermentation. Still, traces of *L. bulgaricus* were found throughout the fermentation. The rate of heat flow curves shows that SC1 was the only culture with a single growth phase, indicating that pH limitation decelerates the growth of *S. thermophilus*, but *L. bulgaricus* was not strong enough to take over the culture. The research by Adamberg et al. [24], showed that *S. thermophilus* St20 was acid sensitive and unable to grow below pH 5.1, which is consistent with the present work.

SC2 has five different species in the culture, but amplicon sequencing does not distinguish between *L. delbrueckii* spp*. Bulgaricus* and *L. delbrueckii* spp*. Lactis* and identifies them as a single species (marked as L. bulgaricus* in Fig. 4). At the beginning of fermentation, *S. thermophilus* was the dominating species, followed by *L. acidophilus* at 10.8%. The minority species were *L. bulgaricus/lactis* and *B. lactis*, with proportions of 1.8% and 0.2%, respectively. *B. lactis* was not detected at later timepoints, indicating that three other species took over the culture during the fermentation process. Interestingly, *L. bulgaricus/lactis*, which has only 1.8% abundance at the beginning, almost disappeared by 6th hour and after that, its proportion in the culture started to increase again, reaching 6.7% and 22.5% after 12 h and 24 h of fermentation, respectively. The same phenomenon occurs in cultures SC3 and SC4, where *S. thermophilus* reaches its maximal abundance by hour 6, after which its proportion begins to decrease, and other species start to take over the culture. At the end of the exponential growth phase, the heat flow increased slightly, creating peaks due to the secondary slow growth of the same three cultures (SC2, SC3, SC4), and after 9 h, the abundance of other populations in the starter consortium started to increase.

*L. paracasei* was initially not detected in the SC4 culture. It first appeared after 3 h of fermentation and was the second dominant species at 17.6% after 24 h. Generally, the species that dominated in the beginning of fermentation was the most abundant population until conditions were no longer favorable. In this starter culture comparison, *S. thermophilus* was the dominant species in all fermented oat drinks. However, in SC2 culture, after 24 h sampling point, *S. thermophilus* relative abundance is lower (75.8%) than at the starting point (81.6%). It seems that the starting conditions were suitable for the growth of *S. thermophilus*, but the limiting factor was the drop in pH, and the other starter cultures were able to slowly increase their proportion in the fermented oat drink. In this type of product development, *S. thermophilus* is the dominant species because the fermentation time is usually around 6–12 h or even less. Studies have shown that during the first hours of fermentation, *S. thermophilus* is the main lactose degrader, while in the later stages of yoghurt production, *L. bulgaricus* increases its abundance [25,26]. Compared to other LAB species, in addition to the dominant *S. thermophilus*, *L. plantarum* was constantly present in the SC3 consortium. This may be because *L. plantarum* originates from plant and is adapted to grow on plant material [27]. The dominance of the two species during fermentation indicates that these bacteria probably did not compete for the same carbon source. While *S. thermophilus* prefers to metabolize mono- and disaccharides, *L. plantarum* is capable of fermenting different fractions from starch, both linear and branched subunits [28]. Luana et al. [11], showed that only *L. plantarum* strains were capable of fermenting oat beverages at +30 °C for 12 h up to a pH of 4.2, whereas drinks with *L. casei* and *L. paracasei* did not reach below a pH of 4.7. Moreover, in oat, barley, or malt-based substrates, *L. plantarum* was the only bacterium maintaining viability of 10^7^ cfu/mL after 24 and 36 h of fermentation, compared to *L. acidophilus* and *L. reuteri* [29]. These observations indicate that *L. plantarum* should be considered as a valuable species for fermentation of plant material next to *S. thermophilus* with excellent acidification capability and high acid tolerance.

### 3.3 Chemical, textural, and sensory evaluation of fermented oat drink

#### 3.3.1 Chemical parameters of fermented oat drink

**Fig. 5:**
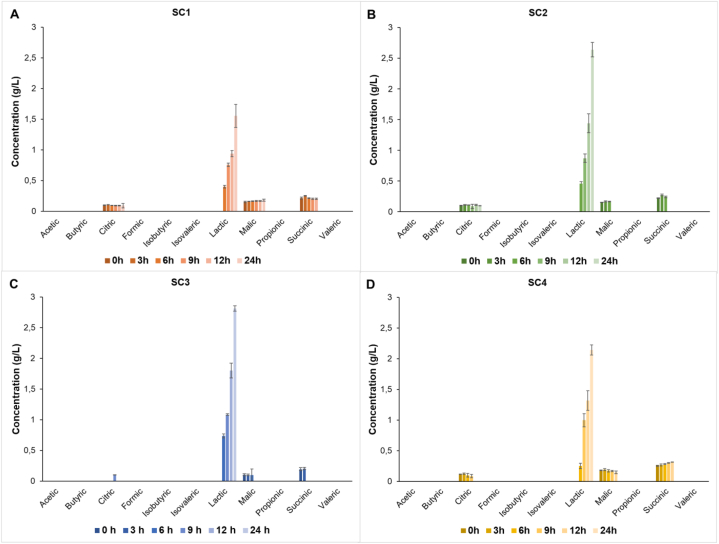
Organic acid concentration (g/L) change during fermentation. Organic acid concentrations for SC1 is shown on graph A, for SC2 on graph B, for SC3 on graph C and for SC4 on graph D. Data represents each sample in the form of mean of biological replicates ± SD (n = 3).

Throughout the study (every 3 h up to 12 h and then after 24 h of fermentation) the concentration of sugars, organic acids and free amino acid was measured (Fig. 5, Supplementary materials – Fig. S.1, Fig. S.2). The initial sugar content was similar in all oat drinks with different starter cultures. The trisaccharide concentration was 6.5 g/L, the disaccharide concentration was 32 g/L, and the glucose concentration was 5 g/L. The results showed that after 24 h, glucose was the only measured sugar source consumed by the starter cultures. Interestingly, the concentration of tri- and disaccharide in some starter cultures even increased, indicating that even longer sugar polymers were probably present in the matrix. Thus, the bacteria likely consumed additional carbon sources besides glucose, increasing tri- and disaccharides residues in the medium. However, *S. thermophilus* cannot metabolize amylose and amylopectin [30,31], preferring primarily monosaccharides. Still, amylolytic activity was observed in some LAB strains, resulting in the release of mono- and disaccharides [29].

The organic acid results from HPLC analysis show a homofermentative process for all starters (Fig. 5). The only acid produced during fermentation was lactic acid. Homolactic fermentation is a redox-neutral process where glucose is metabolized to lactic acid, thus ATP can be produced in glycolysis without accumulating the excess NADH in the cell. The highest concentration of lactic acid was reached at 24 h and from the highest to the lowest concentrations, SC3 produced the most at 2.81 g/L, followed by SC2 at 2.64 g/L, SC4 at 2.14 g/L and SC1 at 1.56 g/L. The uniqueness of SC3 is that it contains *L. plantarum*, a common lactic acid bacterium isolated from plants. The oat drink environment could be most suitable for this microorganism, and therefore lactic acid was produced in higher concentrations. Also, the metagenomic results showed that the appearance of *L. plantarum* increased after 24 h of fermentation. In all starter cultures, the lactic acid concentration increased more than 1.5 times with extra 12 h of fermentation.

None of the starter cultures produced ethanol during fermentation, indicating that the entire process was aerobic [32]. Acetic acid was also not measured with HPLC at different timepoints throughout the fermentation. However, under aerobic conditions, when lipoic acid is not available, *S. thermophilus* requires acetic acid to produce biomass [33]. It could be that the acetic acid was produced by other species (such as *L. paracasei*, *L. plantarum*) and was immediately consumed by *S. thermophilus*. Furthermore, *S. thermophilus* can also utilize lipoic acids, which are unique molecules from both plant and animal tissues that are also present in oats [34]. Lipoic acid is a co-factor of pyruvate dehydrogenase, which catalyzes the reaction from pyruvate to Acetyl-CoA, a key molecule in the breakdown of a carbon source and required for bacterial growth [35]. Some organic acids like citric, malic, and succinic acids were present in small quantities from the beginning, but all were consumed within 12–24 h in all batches. The dominating *S. thermophilus* was not able to utilize malic and succinic acids due to the lack of enzymes in the reductive branch of the TCA cycle. Thus, *S. thermophilus* did not grow during these hours, and probably other lactic acid bacteria consumed aforementioned organic acids. Unfortunately, citric acid was not measured properly in almost all SC3 timepoints, while in the sample this organic acid measurements were below the limit of qualification (LOQ). Still, citrate was depleted by the SC4 starter, containing *L. paracasei*. Analysis of the NCBI protein database reveals that the genomes of *L. plantarum* and *L. paracasei* contain genes encoding enzymes necessary for citric acid degradation.

Fluctuations in free amino acid concentration were measured throughout fermentation (Supplementary materials – Fig. S.2). The results indicate that the highest consumption occurred in 24 h and that free Asn, Asp and Glu concentrations decreased the most for all starter cultures. However, some free amino acids concentrations increased after 24 h of fermentation. An increase in Pro and Ser was detected in all starter cultures. The results are opposite found by Luana et al. [11], where Asp concentration increased and Ser concentration decreased in oat drink fermented with *L. plantarum*. The contradiction can be explained by the fact that Luana et al. [11], used a single *L. plantarum* culture, while the current study used a consortium. The increase in the concentration of some free amino acids was probably caused by the consumption of peptides, since it is energetically feasible to take in peptides of several amino acid long and excrete futile amino acids [36].

#### 3.3.2 Rheological parameters of fermented oat drink

12 h fermented oat drink samples were collected for rheological analysis and amplitude and frequency sweeps were performed; these results are summarized in Table 1. Apart from the control, which was unfermented oat drink sample with G’_LVE_ of 0.59 ± 0.02 Pa and viscoelastic almost liquid-like behavior (G’≤G″, data not shown), fermented oat drink samples had approximately two times higher G’_LVE_ and showed weak gel-like structures (G’>G″). Amplitude sweep analysis showed that among others fermentation with SC4 resulted in most gel-like characteristics. SC4 had the highest structural rigidity and gel strength, since it scored highest values among G’_LVE_ and τ_y_, respectively. Frequency sweep curves also showed a weak gel-like character of all fermented oat drink samples over the whole frequency range indicating the presence of some network structure (data not shown). A lower slope of G′-curve at lower frequencies (Δ log G’/Δ log f) of the sample fermented with SC4 indicates the higher physical long-term stability and a lower tendency to sedimentation. This is in accordance with the information provided by the starter manufacturer, which described the SC4 with an ability to give a higher texture. SC4 consortium contains *L. paracasei* that, according to the NCBI protein database, has enzyme coding genes for EPS production. However, *L. plantarum* that was present in SC3 also produces EPS, but this batch did not have as high viscosity as SC4. The EPS produced by these two species were different and affected the structure of the final fermented product diversely [37,38]. The EPS production by starter culture is crucial for dairy alternative formulation, while this study also showed that unfermented samples had poor texture, were layered and were the first ones to lose their structure. The rheological analysis pairs with sensory analysis results, which showed that SC4 had more viscous mouthfeel.

**Table 1:** Rheological parameters derived from amplitude and frequency sweeps conducted with control (unfermented oat drink) and fermented oat drink samples.

Starter	aG′_LVE_, Pa	bτ_y_, Pa	cΔ log G’/Δ log f, Pa/Hz
Control	0.59 ± 0.02^a^	0.002 ± 0.000^a^	0.11 ± 0.02 ^ac^
SC1	1.09 ± 0.11^b^	0.006 ± 0.005 ^ab^	0.17 ± 0.04^b^
SC2	0.83 ± 0.12 ^ac^	0.003 ± 0.001^a^	0.14 ± 0.02 ^ab^
SC3	1.00 ± 0.16 ^bc^	0.004 ± 0.000 ^ab^	0.17 ± 0.03 ^ab^
SC4	1.39 ± 0.15^d^	0.011 ± 0.006^b^	0.08 ± 0.03^c^

Means with different letters are sign. Different from one another p < 0.05.

a G’_LVE_ – Storage modulus within the linear viscoelastic range.

b Τ_y_ – Yield stress.

c Δ log G’/Δ log f – Slope of storage modulus vs frequency.

#### 3.3.3 Volatile compounds of fermented oat drink

**Fig. 6:**
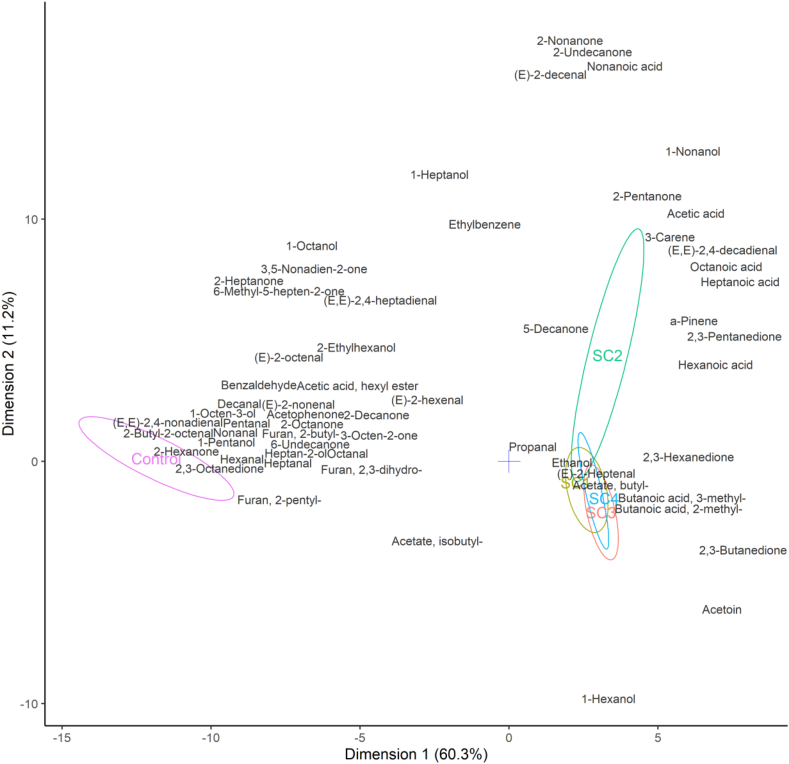
PCA biplot of oat drink (control) and oat drinks fermented with different starter cultures (SC1-4), showing correlations with volatile compounds extracted by HS-SPME. Sample clusters are shown by 95% confidence ellipses. Variables are centered and scaled. Data represents each sample in the form of mean of biological replicates (n = 3).

More than 60 volatile compounds were identified after analyzing the oat drink bases fermented with four different starter cultures. The correlation between volatile compounds in unfermented and fermented oat drink is shown in Fig. 6. Most of the volatile compounds identified belonged to the aldehyde, ketone, acid, furan, and alcohol classes.

Aldehydes, such as pentanal, hexanal, heptanal, octanal, nonanal, decanal and benzaldehyde give sweetness and green notes to non-inoculated drinks in both odor and taste perception. For example, the unfermented drink had a hexanal (grass) concentration of 645 parts per billion (ppb), while the fermented samples had less than 10 ppb (data not shown). Hexanal was reported to be the most significant volatile in dry non-thermal treated oats. It is the result of oxidation of linoleic acid and is also the main volatile component in rancid oat groats [39]. Furthermore, nonanal (fatty, citrus) and decanal (fatty, orange) were only present in trace amounts in the fermented samples but were abundant in the unfermented samples**.** Nonanal is a product of degradation of oleic acid and is derived from fresh, untreated oat groats [39].

Meanwhile, ketones were more prominent in the fermented samples. Concentrations of creamy ketones like diacetyl (2,3-butanedione), acetylproprionyl (2,3-pentanedione), acetylbutyryl (2,3-hexanedione) and acetoin (2-butanone, 3-hydroxy-) were high in all inoculated samples but only traces were present in unfermented samples. The above-mentioned ketones give fermented samples a sweet, round, more balanced flavor. Diacetyl concentrations increased during fermentation, being highest for SC1 and SC4 at 226 ± 2.6 and 223 ± 5.9 ppb, respectively (data not shown). Diacetyl production is inconsistent with the results of [29], who stated that *L. plantarum, L. acidophilus* and *L. reuteri*, could only produce diacetyl in malt-based media, but not in oat and barley media. It could be that other lactic acid bacteria, such as *S. thermophilus*, were required for diacetyl production [40]. SC3 (consortium combination additionally includes *L. plantarum*) and SC4 (consortium additionally includes *L. paracasei*) inoculated beverages had two-fold higher acetoin concentrations compared to the other two starters (data not shown). Furthermore, the concentrations of some unwanted ketones were reduced by fermentation. 2-Heptanone (blue cheese), 2,3-octanedione (herbal), 2-octanone (overripe, moldy) and acetophenone (almond, musty) were present in the inoculated samples at lower concentrations than in the blanks, with one exception where 2-nonanone (green, herbal) concentration was higher in SC2 inoculated samples.

Acids, namely acetic acid (vinegar), 3-methyl- and 2-methylbutanoic acids (cheesy) and hexanoic acid (goat cheese) increased in all fermented samples. According to the GC-MS results, acetic acid has the highest relative intensity of the identified acids. The highest production of acetic acid was detected in drinks inoculated with SC2 and SC3. Sensorially, acetic acid gives a sharp acid flavor, while lactic acid gives a mild sour flavor. Acetic acid in SC2 was 21 ± 1.1 ppb, in SC3 14 ± 1.7 ppb, in SC4 9 ± 0.1 ppb and in SC1 6 ± 0.3 ppb (data not shown). Acetic acid concentrations correlated with the results of sensory analysis of acidic flavor intensity in the decreasing order: SC2>SC3>SC4>SC1. However, no acetic acid was detected in the HPLC analysis. The explanation is that *S. thermophilus* (present in all four starter cultures) consumed significant amounts of acetic acid and the sensitive GC-MS analysis was able to detect traces in the fermented oat drink. Furthermore, the concentrations of hexanoic (cheesy, goat), octanoic (blue cheese) and nonanoic (cheese, dairy) acid increased in all fermented samples. This is in accordance with the results of Salmeron et al. [12], who found that octanoic and nonanoic acids were characteristic of oat drink fermented with *L. plantarum* NCIMB 8826, but were not released during fermentation of wheat, barley, and malt drink.

Concentrations of 2-Butylfuran (wet hay) and 2-Pentylfuran (beany, earthy) were higher in blanks, but decreased five-fold in all fermented samples. 2-Pentylfuran comes from fresh oat groats [39]. Amongst the detected alcohols, green and herbal notes were prevailing. 1-Hexanol (floral, green) was present in high concentrations in all samples. In the fermented samples, 1-Pentanol (green) was 3-fold and 1-Octen-3-ol (mushroom, earthy) was ten-fold lower. The results are consistent with previous studies showing 1-pentanol in fresh oat groats and 1-hexanol and 1-octen-3-ol in hydrated groats [39].

#### 3.3.4 Sensory properties of fermented oat drink

**Fig. 7:**
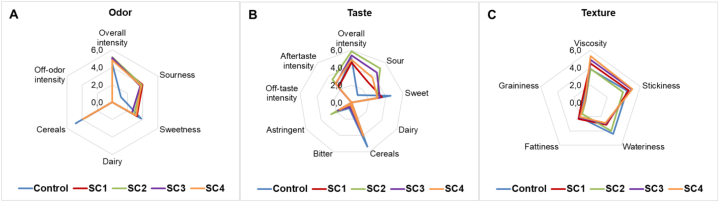
Sensory profile of oat drinks (control and fermented with different starter cultures for 12 h) by odor (A), taste (B), and texture (C). Mean values of assessors are indicated (n = 9).

In this experiment, the optimal time for fermentation to reach a kefir-like product was 12 h, and samples were collected for sensory evaluation at this time point. All fermented oat drink samples had an overall similar odor, taste, and textural properties that differed from the unfermented sample. After fermentation, an increase in sourness of odor and taste was observed (Fig. 7). The sourness of odor increased from 1.1 to 4.0 points and of taste increased from 1.1 to 5.1 points. Simultaneously with the sourness increase, the sweetness decreased slightly. Since the total sugar concentration measured by HPLC was not affected by fermentation, the decrease in sweetness perception may be due to the increase in sourness. Although diacetyl and acetoin concentrations increased during fermentation, all samples were associated with cereals but not dairy products in both odor and taste. Luana et al. [11], showed that fermentation with *L. plantarum* increased mainly sour taste and reduced artificial and earthy notes in fermented oat drinks. None of the samples had off-odors or -flavors. Among the fermented samples, SC2 was the highest and SC1 was the least sour. This is in accordance with the organic acid production and the specifications provided by the manufacture. The fermented drinks had low values in bitterness, while unfermented sample was the most bitter with a value of 0.7. A higher astringency and aftertaste intensity were observed for SC2, which might be related to the higher sourness of the sample.

The viscosity of the fermented samples was higher than of unfermented drink, except for SC2. SC4 was evaluated as the most viscous (Fig. 7). The sensory analysis is consistent with the results of the rheological measurements, which showed that SC4 had the most gel-like structure, and with the information provided by the starter manufacturer, which described SC4 to provide a high texture. Fermented oat drinks had higher stickiness and lower wateriness than the unfermented drink. The samples had low scores in fattiness and there was no graininess. This result is on contrary to the data of Luana et al. [11], who found a change in viscosity in the opposite direction during oat base fermentation. The reduction in thickness during fermentation could be related to the viscosity of the oat base, while 25% of oat flour was used compared to our 16%.

Perceived sourness was compared with the results of titratable acidity (Supplementary materials – Fig. S3). The results were in correlation and consistent with the iCinac and organic acid analysis results. Of the fermented oat beverages, SC2 was the most sour and had the highest acid content, followed by SC3 and SC4, with SC1 being the least sour. Titratable acidity was 0.51, 0.45, 0.38 and 0.3% of lactic acid for SC2, SC3, SC4 and SC1, respectively.

## 4 Conclusion

The global demand for plant-based dairy alternatives is constantly increasing. However, little is known about the fermentation processes of plant-based drinks. The main objective of the present study was to understand and demonstrate the changes in pH, consortium population, metabolism, chemical composition, structural and sensory properties during oat drink fermentation. The oat drinks were fermented with four different vegan starter cultures containing various lactic acid bacteria. Combining isothermal microcalorimetry with 16 S metagenomic analysis showed that a kefir-like product with pH < 4.2 was obtained after 12 h and that *S. thermophilus* was the dominant species throughout the fermentation. In the later stages of fermentation *L. acidophilus, L. plantarum* and *L. paracasei* slowly increased in the fermented oat drink, but never reached the dominant concentration in the beverage. The prevalence of *S. thermophilus* was also indicated by organic acid production, with lactic acid being the most produced metabolite. Concentrations of the most favored volatile compounds such as diacetyl and acetoin increased during fermentation. However, during sensory evaluation, all samples were associated with cereal and not dairy in terms of odor and taste during sensory evaluation. Fermented oat drinks had approximately twice the G’_LVE_ and had a weak gel-like structures (G’>G″) compared to the unfermented oat drink base. Rheological analysis combined with sensory analysis revealed a more viscous mouthfeel for oat drinks fermented with SC4. This comprehensive study shows the growth dynamics of various vegan starter cultures and changes in the composition of bacterial consortium, which in turn affected the sensory and textural formation of the fermented oat drink and helps food manufactures to choose suitable starter cultures for the plant-based dairy alternative production.

## Author contribution statement

Mary-Liis Kütt: Conceived and designed the experiments; Performed the experiments; Analyzed and interpreted the data; Contributed reagents, materials, analysis tools or data; Wrote the paper.

Kaisa Orgusaar, Irina Stulova: Performed the experiments; Analyzed and interpreted the data; Contributed reagents, materials, analysis tools or data; Wrote the paper.

Reimo Priidik, Indrek Morell: Analyzed and interpreted the data; Wrote the paper.

Dmitri Pismennõi, Helen Vaikma, Aili Kallastu, Aleksandra Zhogoleva, Tiina Kriščiunaite: Performed the experiments; Analyzed and interpreted the data; Wrote the paper.

## Data availability statement

Data included in article/supp. Material/referenced in article.

## Declaration of interest's statement

The authors declare no competing interests.

## Funding

This study was conducted as a part of the project of the Innovation Cluster for Plant Proteins (616118790021)and was funded by the Estonian Rural Development Plan (ERDP) for 2014–2020 and the European Agricultural Fund for Rural Development (EAFRD). The European Regional Development Fund (ERDF) and Estonian Research Council provided additional support via project RESTA16.

## Ethical statement

All participants from a pool of highly trained evaluators in the sensory panel gave written consent to take part in the experiment. Participants were informed in advance of the purpose and the procedures of the study. Participants were assured of the confidentiality of their data. Taking part in the given study was voluntary and one could withdraw from the test at any time. Participants were in good health and had no known allergy to the components. Institutional approval for the research is not available due to Estonian requirements for human research. Ethical statement is also included at the end of the manuscript.

## Declaration of competing interest

The authors declare that they have no known competing financial interests or personal relationships that could have appeared to influence the work reported in this paper.
