# Supplementary material for: Starter culture growth dynamics and sensory properties of fermented oat drink
Source: Heliyon. 2023 Apr 25;9(5):e15627. doi: 10.1016/j.heliyon.2023.e15627 (PMC10173617; doi:10.1016/j.heliyon.2023.e15627)
Supplement: Multimedia component 1 [file mmc1.docx]

**Supplementary materials**

**Table S.1** Growth characteristics of starter cultures in IMC at 40°C*

| **Starter** | ^A^**µ_max_, h^-1^** | ^B^**P_max_, µW** | ^C^**t_Pmax_, h** | ^D^**Q_exp_, J/mL** | ^E^**Q_tot_, J/mL** |
| --- | --- | --- | --- | --- | --- |
| **SC1** | 1.66 ± 0.02 | 73.06 ± 1.91 | 3.81 ± 0.10 | 0.30 ± 0.00 | 1.29 ± 0.04 |
| **SC2** | 1.55 ± 0.03 | 75.53 ± 2.10 | 3.39 ± 0.05 | 0.32 ± 0.03 | 2.19 ± 0.05 |
| **SC3** | 1.53 ± 0.02 | 87.96 ± 0.73 | 3.03 ± 0.05 | 0.31 ± 0.03 | 1.94 ± 0.03 |
| **SC4** | 1.40 ± 0.03 | 70.36 ± 2.06 | 4.42 ± 0.00 | 0.31 ± 0.03 | 1.63 ± 0.08 |

*Data are the means of biological replicates ± SD (n=3).

^A^Maximal calorimetric growth rate (μ_max_) in the first exponential growth phase

^B^Maximum heat flow (P_max_)

^C^The time of maximum heat production rate (t_Pmax_)

^D^Heat evolved during the first exponential phase (Q_exp_)

^E^Total heat produced during the whole fermentation (Q_tot_)


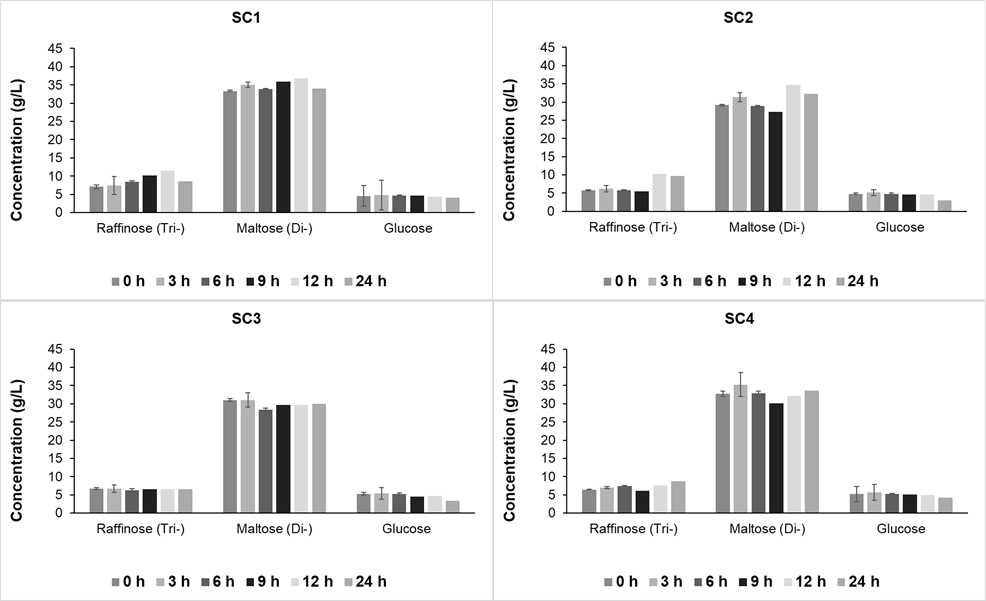


**Fig. S.1.** Sugar concentration (g/L) change during fermentation. Tri- and disaccharides are indicated as sum, where either raffinose or maltose was used as marker, respectively. Data represents each sample in the form of mean of biological replicates ± SD (n=3).


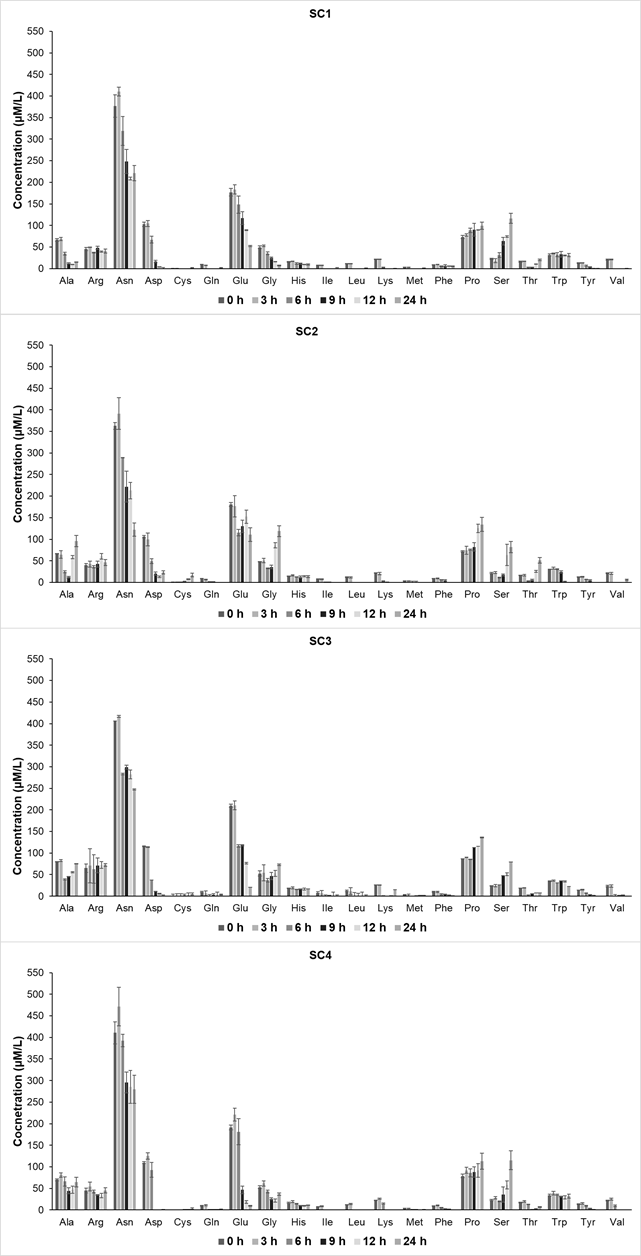


**Fig. S.2.** Free amino acid concentration (μM/L) change during fermentation. Data represents each sample in the form of mean of biological replicates ± SD (n=3).

**Fig. S.3.** Comparison of sourness. Columns indicate the results obtained from sensory evaluation. Black dots indicate the sourness results obtained with total titratable acid analysis. Lactic acid data represents each sample in the form of mean of biological replicates ± SD (n=3).
