# Supplementary material for: Starter culture growth dynamics and sensory properties of fermented oat drink
Source: Heliyon. 2023 Apr 25;9(5):e15627. doi: 10.1016/j.heliyon.2023.e15627 (PMC10173617; doi:10.1016/j.heliyon.2023.e15627)
Supplement: Multimedia component 2 [file mmc2.pdf]

APPEARANCE / VÄLIMUS

Colour intensity / värvi intensiivsus

0	1	2	3	4	5	6	7	8	9
None	Very weak				Moderate				Very strong

Colour evenness / värvi ühtlus

0	1	2	3	4	5	6	7	8	9
None	Very weak				Moderate				Very strong

Glossiness / läikivus

0	1	2	3	4	5	6	7	8	9
None	Very weak				Moderate				Very strong

Additional comments / lisakommentaarid

ODOR / LÕHN

Overall intensity / üldine intensiivsus

0	1	2	3	4	5	6	7	8	9
None	Very weak				Moderate				Very strong

Sourness / hapusus

0	1	2	3	4	5	6	7	8	9
None	Very weak				Moderate				Very strong

Sweetness / magusus

0	1	2	3	4	5	6	7	8	9
None	Very weak				Moderate				Very strong

Dairy / piim

0	1	2	3	4	5	6	7	8	9
None	Very weak				Moderate				Very strong

Cereals / teraviljad

0	1	2	3	4	5	6	7	8	9
None	Very weak				Moderate				Very strong

Off-odour intensity / kõrvallõhna intensiivsus

0	1	2	3	4	5	6	7	8	9
None	Very weak				Moderate				Very strong

Additional comments / lisakommentaarid

TASTE / MAITSE

Overall intensity / üldine intensiivsus

0 1 2 3 4 5 6 7 8 9
None Very weak Moderate Very strong

Sour / hapu

0 1 2 3 4 5 6 7 8 9
None Very weak Moderate Very strong

Sweet / magus

0 1 2 3 4 5 6 7 8 9
None Very weak Moderate Very strong

Dairy / piim

0 1 2 3 4 5 6 7 8 9
None Very weak Moderate Very strong

Cereals / teraviljad

0 1 2 3 4 5 6 7 8 9
None Very weak Moderate Very strong

Bitter / kibe

0 1 2 3 4 5 6 7 8 9
None Very weak Moderate Very strong

Astringent / kootav

0 1 2 3 4 5 6 7 8 9

None Very weak Moderate Very strong

Off-taste intensity / kõrvalmaitse intensiivsus

0 1 2 3 4 5 6 7 8 9

None Very weak Moderate Very strong

Aftertaste intensity / järelmaitse intensiivsus

0 1 2 3 4 5 6 7 8 9

None Very weak Moderate Very strong

Additional comments / lisakommentaarid

TEXTURE / TEKSTUUR

Viscosity / viskoossus
(Spoon)

0 1 2 3 4 5 6 7 8 9

None Very weak Moderate Very strong

Stickiness (sliminess) / kleepuvus

0 1 2 3 4 5 6 7 8 9

None Very weak Moderate Very strong

Wateriness / vesisus

0 1 2 3 4 5 6 7 8 9

None Very weak Moderate Very strong

Fattiness / rasvasus

0 1 2 3 4 5 6 7 8 9

None Very weak Moderate Very strong

Graininess / teralisus

0 1 2 3 4 5 6 7 8 9

None Very weak Moderate Very strong

Additional comments / lisakommentaarid
